# Identification of Semantically Similar Sentences in Clinical Notes: Iterative Intermediate Training Using Multi-Task Learning

**DOI:** 10.2196/22508

**Published:** 2020-11-27

**Authors:** Diwakar Mahajan, Ananya Poddar, Jennifer J Liang, Yen-Ting Lin, John M Prager, Parthasarathy Suryanarayanan, Preethi Raghavan, Ching-Huei Tsou

**Affiliations:** 1 IBM Research Yorktown Heights, NY United States; 2 National Taiwan University Taipei Taiwan; 3 Formerly IBM Research Yorktown Heights, NY United States

**Keywords:** electronic health records, semantic textual similarity, natural language processing, multi-task learning, transfer learning, deep learning

## Abstract

**Background:**

Although electronic health records (EHRs) have been widely adopted in health care, effective use of EHR data is often limited because of redundant information in clinical notes introduced by the use of templates and copy-paste during note generation. Thus, it is imperative to develop solutions that can condense information while retaining its value. A step in this direction is measuring the semantic similarity between clinical text snippets. To address this problem, we participated in the 2019 National NLP Clinical Challenges (n2c2)/Open Health Natural Language Processing Consortium (OHNLP) clinical semantic textual similarity (ClinicalSTS) shared task.

**Objective:**

This study aims to improve the performance and robustness of semantic textual similarity in the clinical domain by leveraging manually labeled data from related tasks and contextualized embeddings from pretrained transformer-based language models.

**Methods:**

The ClinicalSTS data set consists of 1642 pairs of deidentified clinical text snippets annotated in a continuous scale of 0-5, indicating degrees of semantic similarity. We developed an iterative intermediate training approach using multi-task learning (IIT-MTL), a multi-task training approach that employs iterative data set selection. We applied this process to bidirectional encoder representations from transformers on clinical text mining (ClinicalBERT), a pretrained domain-specific transformer-based language model, and fine-tuned the resulting model on the target ClinicalSTS task. We incrementally ensembled the output from applying IIT-MTL on ClinicalBERT with the output of other language models (bidirectional encoder representations from transformers for biomedical text mining [BioBERT], multi-task deep neural networks [MT-DNN], and robustly optimized BERT approach [RoBERTa]) and handcrafted features using regression-based learning algorithms. On the basis of these experiments, we adopted the top-performing configurations as our official submissions.

**Results:**

Our system ranked first out of 87 submitted systems in the 2019 n2c2/OHNLP ClinicalSTS challenge, achieving state-of-the-art results with a Pearson correlation coefficient of 0.9010. This winning system was an ensembled model leveraging the output of IIT-MTL on ClinicalBERT with BioBERT, MT-DNN, and handcrafted medication features.

**Conclusions:**

This study demonstrates that IIT-MTL is an effective way to leverage annotated data from related tasks to improve performance on a target task with a limited data set. This contribution opens new avenues of exploration for optimized data set selection to generate more robust and universal contextual representations of text in the clinical domain.

## Introduction

### Background

**Figure 1:**
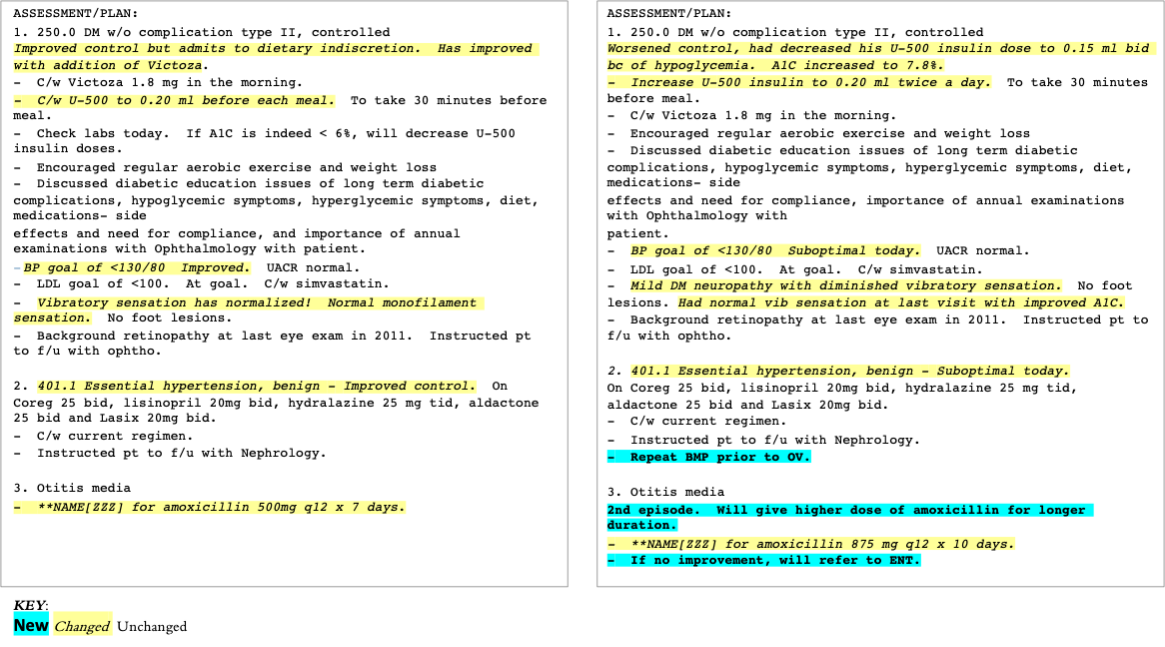
Two sample clinical notes for the same patient from consecutive visits. Plain text indicates same content between 2 notes; italics (yellow highlight) indicate the content that has been modified, and bold (blue highlight) indicates new content in the second note.

The wide adoption of electronic health records (EHRs) has led to clinical benefits with increased efficiency and financial benefits [1]. Although electronic documentation has greatly improved the legibility and accessibility of clinical documentation, the use of templates and copy-paste during note generation has inadvertently introduced unnecessary, redundant, and potentially erroneous information (ie, note bloat), resulting in decreased readability and functional usability of the generated clinical notes [2-5]. A previous study [6] on 23,630 clinical notes identified that in a typical note, only 18% of the text was manually entered, whereas 46% was copied and 36% imported. This problem of note bloat not only increases physician cognitive burden [7] but also becomes a challenge for the secondary use of EHRs in clinical informatics [8]. Figure 1 illustrates this challenge with an example of 2 sample clinical notes from the same patient from consecutive visits; blue and yellow highlighted text indicate content that have been added or modified, respectively, whereas the plain unhighlighted text indicates information that is the same across clinical notes.

One way to minimize data redundancy and highlight new information in unstructured clinical notes can be to compute the semantic similarity between clinical text snippets. This process of measuring the degree of semantic equivalence between clinical text snippets is known as clinical semantic textual similarity [9]. As semantic textual similarity (STS) is a foundational language understanding problem, successful modeling of this task may help improve other higher-level applications in the clinical domain [9], such as clinical question answering with evidence-based retrieval, clinical text summarization, semantic search, conversational systems, and clinical decision support.

The 2019 National NLP Clinical Challenges (n2c2)/Open Health Natural Language Processing Consortium (OHNLP) track on clinical semantic textual similarity (ClinicalSTS) [10] was organized to tackle this specific task: given a pair of clinical text snippets, assign a numerical score from 0 to 5 to indicate the degree of semantic similarity. This is an extension of a previous challenge from BioCreative/OHNLP 2018 ClinicalSTS [11,12] that was inspired by the Semantic Evaluation (SemEval) semantic textual similarity (STS) shared tasks [13-18], which have been organized since 2012 in the general domain.

Pretrained language models have been shown to be effective for achieving state-of-the-art results on many general and clinical domain natural language processing (NLP) tasks [19], including STS. However, when the target domain differs substantially from the pretraining corpus, the contextualized embeddings may be ineffective for the target task. Furthermore, when the amount of training data are limited, as is common for clinical NLP tasks, fine-tuning experiments are potentially brittle and rely on the pretrained encoder parameters to be reasonably close to an ideal setting for the target task [20]. A previous study has shown that small training data sets can significantly benefit from an intermediate training step [20]. In a complementary work, multi-task learning (MTL) [21] has been shown to be effective in leveraging supervised data from multiple related tasks for a target task. Furthermore, it has been observed that MTL and language model pretraining are complementary technologies [21].

On the basis of these observations, we present a novel methodology that iteratively performs intermediate training of a pretrained language model in an MTL setup using related data-rich tasks. In this iterative process, related data sets were purposefully selected to induce representative knowledge of the target task. In addition, we evaluated the impact of combining multiple transformer-based language models pretrained on diverse corpora. Our system ranked first in the 2019 n2c2/OHNLP ClinicalSTS challenge, achieving state-of-the-art results.

### Relevant Literature

STS is defined as the comparison of a pair of text snippets, approximately one sentence in length, resulting in a numerical score that takes a value on a continuous scale of 0 to 5, indicating degrees of semantic similarity [9,18]. STS, along with paraphrase detection and textual entailment, is a form of semantic relatedness task. Paraphrase detection is the identification of sentences that are semantically identical [22], whereas textual entailment is the task of reasoning if one text snippet can be inferred from another [23-25]. STS is more similar to paraphrase detection because of the symmetricity of the relationship, as compared with entailment, which is asymmetric. However, unlike paraphrase detection, STS expands on the binary output scoring in paraphrase detection to capture gradations of relatedness.

Early research on STS, in both the general and clinical domains, focused on lexical semantics, basic syntactic similarity, surface form matching, and alignment-based methods [26-28]. The overarching theme behind these methods is the identification, alignment, and scoring of semantically related words and phrases and aggregating their scores. However, the absence of a principled way of combining the topological and semantic information led to the construction of sentence representations by building a linear composition of the distributed representations of individual words [29-32]. Although these techniques were an improvement over traditional approaches, they fell short as they did not take the surrounding context into account while generating distributed representations.

Early attempts at building richer representations that encode several linguistic aspects of a sentence for computing similarity included paragraph vectors [33-36], word embedding weighting and principal component removal [37], and convolutional deep structured semantic model [38,39]. However, recent studies on pretrained language models have achieved a breakthrough in sentence representation learning [19,40,41]. Bidirectional encoder representations from transformers (BERT) build upon the ideas from the transformer [42] to construct rich sentence representations and has achieved state-of-the-art results on many general and clinical domain NLP tasks [24,43]. In this process, a transformer-based model is first pretrained on large corpora to learn universal language representations and is then fine-tuned with a task-specific output layer for the target task. BERT has been adapted to biomedical (bidirectional encoder representations from transformers for biomedical text mining [BioBERT]) [44] and clinical (bidirectional encoder representations from transformers on clinical text mining [ClinicalBERT]) domains [45,46].

The performance of BERT and its domain-specific variants could be further improved through MTL. MTL [47] refers to training a model simultaneously for multiple related tasks, and MTL benefits from a regularization effect by alleviating overfitting to a specific task, thus making the learned representations universal across tasks. Supplementary training on intermediate tasks refers to the second stage of pretraining of a model, with data-rich intermediate supervised tasks. Recent studies, such as multi-task deep neural networks (MT-DNN) [21] and supplementary training on intermediate labeled-data tasks [20], show that the use of MTL and intermediate pretraining generates more robust and universal learned representations, resulting in better domain adaptation with fewer in-domain labels.

The winning systems in ClinicalSTS 2018 challenge [48] and SemEval 2017 [49] built upon a combination of approaches referenced earlier in this section. In general, they employed ensembled feature engineering methods (random forest, gradient boosting, and XGBoost) with features based on n-gram overlap, edit distance, longest common prefix/suffix/substring, word alignments [50,51], summarization and machine translation evaluation metrics, and deep learning [36,52]. In contrast to these systems, our study builds upon the modern neural approaches referenced earlier. Specifically, our system implements MTL and supplementary training on intermediate labeled tasks with ClinicalBERT to achieve state-of-the-art performance on the ClinicalSTS 2019 task. Following the demonstration of our system at the 2019 n2c2/OHNLP challenge presentation, additional systems leveraging MTL in ClinicalBERT [53,54] have been implemented with promising results.

## Methods

### Data Set

The 2019 ClinicalSTS data set was prepared by the n2c2/OHNLP challenge organizers from sentences collected from clinical notes in the Mayo Clinic’s clinical data warehouse. Candidate sentence pairs were then generated using an average value ≥0.45 of surface lexical similarity methods, namely, Ratcliff/Obershelp [55], cosine similarity, and Levenshtein distance. This resulted in 2054 pairs, of which 1642 were released as the training set and the remaining 412 were held by the organizers for testing. Protected health information was removed using a mix of frequency filtering approach [56] and manual review process. Each sentence pair was independently reviewed by 2 clinical experts and scored on a scale of 0 to 5 based on their semantic equivalence (0 for no semantic equivalence to 5 for complete semantic equivalence). Interannotator agreement was 0.6 based on weighted Cohen kappa. The averaged score between the 2 annotators was used as the gold standard. Table 1 presents a few examples from the data set.

We split the provided training data set of 1642 sentence pairs into 75.03% (1232/1642), 14.98% (246/1642), and 9.99% (164/1642) to form our train, validation, and internal test data sets, respectively.

**Table 1:** Sample sentence pairs and annotations from the clinical semantic textual similarity data set.

Ground truth^a^		Score	Observations	
Sentence 1	Sentence 2		Domain dependence	Comments
“The patient was taken to the *PACU*^b^ in a stable condition.”	“The patient was taken to the *post anesthesia care unit* postoperatively for recovery.”	5.0	Domain specific	Clinical abbreviations
“*Albuterol [PROVENTIL/VENTOLIN] 90 mcg/Act HFA*^c^ *Aerosol 1-2 puffs by inhalation every 4 hours as needed*.”	“*Ipratropium-Albuterol [COMBIVENT] 18-103 mcg/Actuation Aerosol 2 puffs by inhalation two times a day as needed*”	3.5	Domain specific	Medication instruction parsing
“Cardiovascular assessment findings include *heart rate normal, atrial fibrillation with controlled ventricular response*.”	“Cardiovascular assessment findings include *heart rate, first degree AV*^d^ *Block*.”	3.0	Domain specific	Medical concept similarity and medical concept mapping
“He was *prepped and draped in the standard* fashion.”	“The affected shoulder was *prepared and draped with the usual* sterile technique.”	3.0	Domain independent	Alignment
“Musculoskeletal: *Positive* for gait problem, joint swelling and extremity pain.”	“Musculoskeletal: *Negative* for back pain, myalgias and extremity pain.”	1.5	Domain independent	Assertion classification (polarity)

^a^Italics indicate the phrases within each sentence which correspond to the observations.

^b^PACU: post anesthesia care unit.

^c^HFA: hydrofluoroalkane.

^d^AV: atrioventricular.

Analysis of this data set revealed 2 characteristics that we consider in our approach to this task. First, the lack of sufficient training data makes it difficult to train robust machine learning models using only the given training data. Second, clinical semantic similarity relies on both domain-specific (eg, clinical abbreviation expansion, medical concept detection, and medical concept normalization) and domain-independent (eg, assertion classification and alignment detection) aspects, as demonstrated by the sample sentence pairs in Table 1. For the first sentence pair, a domain-specific understanding of PACU as an abbreviation for post anesthesia care unit is necessary to infer the high semantic equivalence. For the fourth sample sentence pair, domain-independent understanding of the difference in polarity between Positive and Negative is necessary to infer the low similarity equivalence.

To address the lack of sufficient training data and leverage the domain-specific and domain-independent aspects of clinical semantic similarity, we propose an approach that combines the following:

- an iterative intermediate multi-task training step for effective transfer learning employing other related annotated data sets
- an ensemble module that combines language models pretrained on both domain-specific and domain-independent data sets and also incorporates other features.

### Iterative Intermediate Training Using MTL

We performed iterative multi-task training on a transformer-based language model using annotated data sets from related tasks to induce representative knowledge of the target task. With each iteration, annotated data sets from related tasks were added or removed. Following data set selection, the language model was then trained using MTL on the selected data sets, fine-tuned on the target task, and its results were evaluated and error analysis was performed to determine the data set selection for the next iteration. We refer to this entire process as iterative intermediate training using multi-task learning (IIT-MTL).

**Figure 2:**
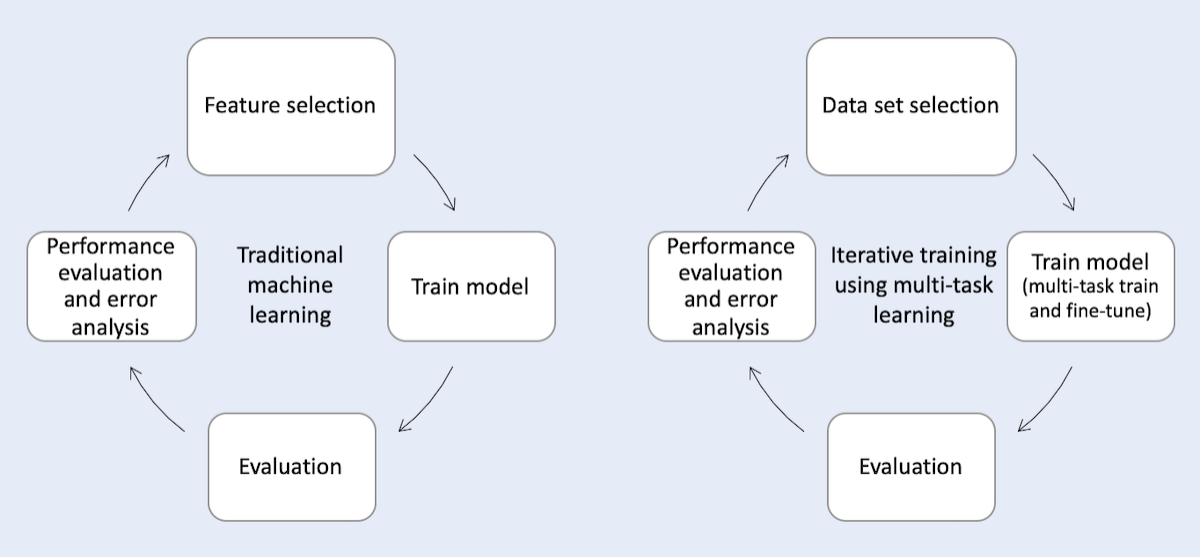
Comparison of traditional machine learning approach (left), where performance evaluation and error analysis lead to feature selection, and our proposed iterative training using multi-task learning approach (right), where performance evaluation and error analysis lead to data set selection.

IIT-MTL is analogous to traditional feature-based machine learning methodologies, where performance evaluation and error analysis lead to feature selection used to train the model. In IIT-MTL, instead of feature selection, data set selection is employed to select data sets. Figure 2 presents IIT-MTL compared with the traditional machine learning approach.

For the ClinicalSTS task, ClinicalBERT was used as our base model as it was pretrained on a clinical corpus and provides clinically specific contextual embeddings most suited to our task. Through IIT-MTL, a refined clinical domain-specific language model, IIT-MTL on ClinicalBERT (IIT-MTL-ClinicalBERT), is obtained that has been iteratively tuned for high performance on the ClinicalSTS task.

In the following sections, we present each step of IIT-MTL as applied to the ClinicalSTS task: (1) the data set selection process, including details of each iteration and data sets used; (2) the MTL architecture with the task-specific layers considered during the iterative process; and (3) fine-tuning on the target task.

### Data Set Selection

For effective performance on the target ClinicalSTS task, we not only trained our model using MTL as an intermediate step but also iteratively selected the data sets employed during this process based on error analysis of the performance on the target task. The selection of complementary data sets is critical to this process as it significantly impacts the contextual representations in the final model.

Several publicly available data sets were considered in these iterations, including Semantic Textual Similarity Benchmark (STS-B) [18], Recognizing Question Entailment (RQE) [57], natural language inference data set for the clinical domain (MedNLI) [24], and Quora Question Pairs (QQP) [58]. STS-B consists of 8.6 K sentence pairs drawn from news headlines, video and image captions, and natural language inference data, each annotated with a score of 0 to 5 to indicate the degree of semantic equivalence. RQE consists of 8.9 K pairs of clinical questions, each annotated with a binary value to indicate entailment (or lack of) between the 2 questions. MedNLI consists of 14 K sentences extracted from clinical notes in the Medical Information Mart for Intensive Care (MIMIC-III) database [59], with each sentence pair annotated as either entailment, neutral, or contradiction. QQP consists of 400 K pairs of questions extracted from the Quora question-and-answer website, each annotated with a binary value to indicate the similarity (or lack of) between the 2 questions. We created 2 additional data sets for use in IIT-MTL for ClinicalSTS: a sentence topic-based data set (Topic) and a medication named entity recognition data set (MedNER). Topic was created on sentences within the ClinicalSTS data set, where each sentence was manually annotated with a label from a predefined list of topics (eg, MED, SIGNORSYMPTOM, EXPLAIN, and OTHER). MedNER was autogenerated using a medication extraction tool [60] on 1000 randomly selected clinical notes in the MIMIC-III database to recognize medications and its related artifacts (eg, strength, form, frequency, route, dosage, and duration). A summary of all data sets used is presented in Table 2, with additional details provided in Multimedia Appendix 1 [10,18,24,57,59-62].

**Table 2:** Data sets used in multi-task learning.

Data set	Task	Domain	Size	Example
STS-B^a^	Sentence pair similarity	General	8600	Sentence 1: “A young child is riding a horse”; Sentence 2: “A child is riding a horse”; Similarity: 4.75
RQE^b^	Sentence pair classification	Biomedical	8900	Sentence 1: “Doctor X thinks he is probably just a normal 18 month old but would like to know if there are a certain number of respiratory infections that are considered normal for that age”; Sentence 2: “Probably a normal 18 month old but how many respiratory infections are normal”; Ground truth: entailment
MedNLI^c^	Sentence pair classification	Clinical	14,000	Sentence 1: “Labs were notable for Cr 1.7 (baseline 0.5 per old records) and lactate 2.4”; Sentence 2: “Patient has normal Cr”; Ground truth: contradiction
QQP^d^	Sentence pair classification	General	400,000	Sentence 1: “Why do rockets look white?”; Sentence 2: “Why are rockets and boosters painted white?”; Ground truth: 1
Topic	Sentence classification	Clinical	1,300,000	Sentence: “Negative for difficulty urinating, pain with urination, and frequent urination”; Ground truth: SIGNORSYMPTOM
MedNER^e^	Token-wise classification	Clinical	15,000	Sentence: “he developed respiratory distress on the AM^f^ of admission, cough day PTA^g^, CXR^h^ with B/L^i^ LL^j^ PNA^k^, started ciprofloxacin and levofloxacin”; Ground truth: ciprofloxacin [DRUG] levofloxacin [DRUG]

^a^STS-B: semantic textual similarity benchmark.

^b^RQE: Recognizing Question Entailment.

^c^MedNLI: natural language inference data set for the clinical domain.

^d^QQP: Quora Question Pairs.

^e^MedNER: medication named entity recognition.

^f^AM: morning.

^g^PTA: prior to admission.

^h^CXR: chest x-ray.

^i^B/L: bilateral.

^j^LL: left lower.

^k^PNA: pneumonia.

We established 2 baselines by fine-tuning 2 pretrained language models, BERT and ClinicalBERT, on the target ClinicalSTS task. Using the stronger baseline of ClinicalBERT, a total of 5 iterations were performed in IIT-MTL for the ClinicalSTS task. The selection of data sets for each iteration was decided based on our understanding of the ClinicalSTS task and error analysis of the results of the previous iteration. The data set selection for each iteration is detailed as follows. For each iteration, D indicates the set of data sets used for multi-task training, following which the model is further fine-tuned to the target ClinicalSTS task and evaluated before the next iteration.

- *Iteration 1: D={STS-B}*: STS-B was employed for multi-task training because it conforms to the same task (STS) in the general domain.
- *Iteration 2: D={STS-B, RQE, MedNLI}*: Next, we added RQE and MedNLI, which are sentence pair classification tasks in the clinical domain, and, hence, are similar to our target task from a domain perspective.
- *Iteration 3: D={STS-B, RQE, MedNLI, Topic}*: Analysis of the output from iteration 2 showed that sentence pairs on different topics within ClinicalSTS express similarity in different ways. Thus, we created and added the Topic data set.
- *Iteration 4: D={STS-B, RQE, MedNLI, Topic, MedNER}*: Analysis of the output from iteration 3 showed that medication instruction sentences (eg, “Tylenol tablet 2 tablets by mouth as needed.”) were the worst performing sentence pairs. To induce medication-related knowledge, we created and added the MedNER data set to the mix.
- *Iteration 5*: D=*{STS-B, RQE, MedNLI, Topic, MedNER, QQP}*: QQP was added in our final iteration as it is a sentence pair classification task, although in the general domain.

The final set of data sets used in the model for the ClinicalSTS task (IIT-MTL-ClinicalBERT) was determined based on the performance analysis of each iteration.

### Intermediate MTL Architecture

**Figure 3:**
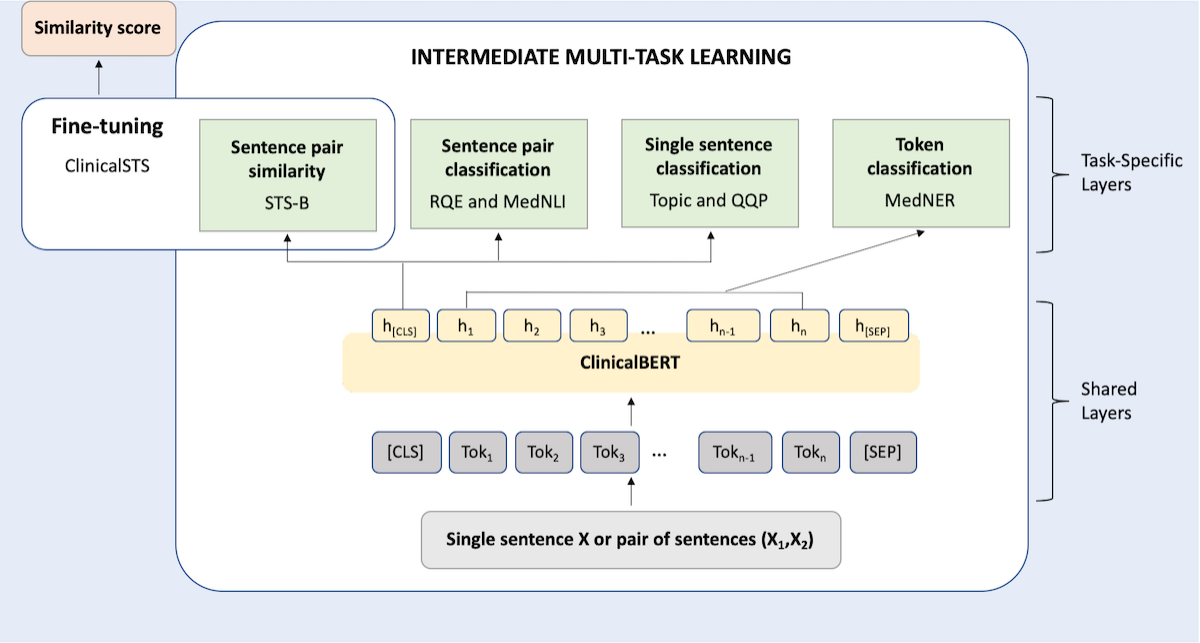
Intermediate multi-task learning and fine-tuning architecture. ClinicalSTS: clinical semantic textual similarity; STS-B: semantic textual similarity benchmark; RQE: recognizing question entailment; MedNLI: natural language inference data set for the clinical domain; QQP: Quora question pairs; MedNER: medication named entity recognition data set; ClinicalBERT: bidirectional encoder representations from transformers on clinical text mining.

The architecture of our intermediate MTL setup is shown in Figure 3 and is based on the process specified in the study by Liu et al [21].

The lower shared layers are based on BERT-base architecture [19], whereas the higher segregated layers represent task-specific outputs. The task-specific layers correspond to the data sets selected during the data set selection.

The input can either be a single sentence (X) or a pair of sentences (X_1_, X_2_) delimited with the separating token ([SEP]). All input texts are tokenized using WordPieces [63] and truncated to spans no longer than 512 tokens. Following this, tokens are added to the start ([CLS]) and end ([SEP]) of the input. In the shared layers, a lexicon encoder converts the input into a sequence of input embedding vectors, one for each token. Next, a transformer encoder captures the contextual information and generates a sequence of contextual embeddings. This semantic representation is shared across all tasks and feeds into multiple lightweight task-specific architectures, each implementing a different task objective. In the training phase, we fine-tuned the shared layers along with task-specific layers using the multi-task objectives, detailed below:

- *Sentence Pair Similarity*: Suppose h_[CLS]_ is the contextual embedding of [CLS] for input sentence pair (X_1_, X_2_) and w_SPS_ is a task-specific parameter vector. We utilized a fully connected layer to compute the similarity score 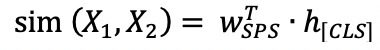 , where 
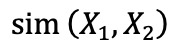
 is a real value of range (−∞, ∞). We use the mean squared error as the objective function:

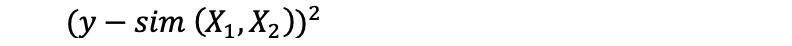


     where y is the similarity score for the sentence pair.

- *Single Sentence Classification*: Suppose h_[CLS]_ is the contextual embedding of [CLS] for input sentence X and w_SSC_ is a task-specific parameter vector. The probability that X is labeled as class c is predicted by logistic regression with softmax:

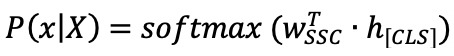


     This task is trained using the cross-entropy loss as the objective:


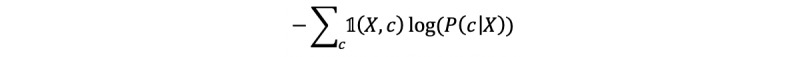


     where 
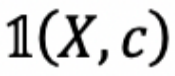
 is the binary indicator (0 or 1) if the class label c is the correct classification for X.

- *Sentence Pair Classification*: Suppose h_[CLS]_ is the contextual embedding of [CLS] for sentence pair (X_1_, X_2_) and w_SPC_ is a task-specific parameter vector. As the two sentences are packed together, we can predict that the relation R between X_1_ and X_2_ is given as 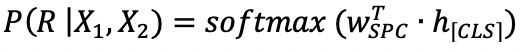 similar to single sentence classification. We trained the task using the cross-entropy loss as specified previously

- *Token Classification*: Suppose h_[1:n]_ is the contextual embedding for tokens Tok _[1:n]_ in packed sentence pair (X_1_, X_2_) and w_TC_ is a task-specific parameter vector. The token classification is trained using a per-entity linear classifier, where the probability that Tok_[j]_ labeled as class c is predicted by logistic regression with softmax: 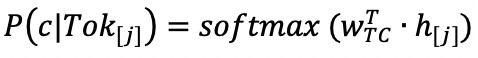. Here, 
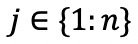
. This task is trained using the cross-entropy loss as specified previously.

The process for training our intermediate MTL architecture is demonstrated in Textbox 1. We initialized the shared layers of our architecture with the parameters of the pretrained ClinicalBERT [46]. The task-specific layers were randomly initialized. We jointly refer to them as θ. Next, we created equal-sized subsamples (mini-batches) from each data set. For every epoch, a mini-batch b_t_ was selected (from each of the MTL data sets detailed previously), and the model was updated according to the task-specific objective for task t. We used the mini-batch–based stochastic gradient descent to update the parameters. A detailed explanation of the training parameters is provided in Multimedia Appendix 2 [19,21,63-65].

**Multi-task learning algorithm.**

Initialize model parameters *θ*

Create E by merging mini-batches (b_t_) for each data set in D

for epoch in 1,2,….., epoch_max_ do

     Shuffle E

     for b_t_ in E do

          Compute loss: *L (θ)* based on task *t*;

          Compute gradient: *∇(θ)*

          Update model: *θ=θ−η∇(θ)*

     end

end

### Fine-Tuning

After multi-task training, we fine-tuned the model on the target ClinicalSTS task. As ClinicalSTS is a sentence similarity task, we fine-tuned the sentence pair similarity task-specific layer of the multi-task architecture (Figure 3) to train the model using the ClinicalSTS data set. The predictions on the internal test data set were evaluated, which drove the data set selection process. A detailed explanation of the training parameters is provided in Multimedia Appendix 2.

### Ensemble Module

To induce both domain-specific and domain-independent aspects of clinical semantic similarity, we leveraged other pretrained language models in addition to IIT-MTL-ClinicalBERT in the ensemble module. During this process, we fine-tuned other pretrained language models on the target task, ensembled their predictions with predictions from IIT-MTL-ClinicalBERT (which was already fine-tuned during IIT-MTL), and then incorporated additional similarity features. In the following sections, we describe the (1) language models used, (2) additional similarity features incorporated, and (3) different ensembling techniques explored.

### Language Models

A total of 4 language models were used in our ensemble module: IIT-MTL-ClinicalBERT, BioBERT [44], MT-DNN [21], and robustly optimized BERT approach (RoBERTa) [66]. IIT-MTL-ClinicalBERT, the output of IIT-MTL, was derived from ClinicalBERT [46], and therefore, it provided clinical domain-specific contextual embeddings. To provide contextual representations from a similar but slightly different domain, we used BioBERT, which is also BERT-based but has been further pretrained on the biomedical corpus. To account for the domain-independent aspects of clinical semantic similarity, we used language models from the general domain, specifically RoBERTa and MT-DNN. RoBERTa is based on BERT but has been optimized for better performance, whereas MT-DNN leverages large amounts of cross-task data, resulting in more generalized and robust text representations. We selected RoBERTa and MT-DNN for use in our ensemble module because at the time of the 2019 n2c2/OHNLP challenge, they achieved state-of-the-art results on multiple tasks similar to ClinicalSTS, including STS-B [43], Multi-Genre Natural Language Inference [23], Question answering Natural Language Inferencing [67], and Recognizing Textual Entailment [68]. Table 3 presents an overview of the language models used in our experiments.

**Table 3:** Pretrained language models used in the ensemble module and their training corpora.

Language model	Corpora for language model pretraining	Domain
MT-DNN^a^	Wikipedia+BookCorpus	General
RoBERTa^b^	Wikipedia+BookCorpus+CC-News+OpenWebText+Stories	General
BioBERT^c^	Wikipedia+BookCorpus+PubMed+PMC^d^	Biomedical
IIT-MTL-ClinicalBERT^e^	Wikipedia+BookCorpus+MIMIC-III^f^	Clinical

^a^MT-DNN: multi-task deep neural networks.

^b^RoBERTa: robustly optimized bidirectional encoder representations from transformers approach.

^c^BioBERT: bidirectional encoder representations from transformers for biomedical text mining.

^d^PMC: PubMed Central

^e^IIT-MTL-ClinicalBERT: iteratively trained using multi-task learning on ClinicalBERT.

^f^MIMIC-III: Medical Information Mart for Intensive Care.

### Other Similarity Features

Under the hypothesis that aggregating similarity metrics from different perspectives could help further boost performance, we incorporated additional string similarity features to our ensembled model. On the basis of the observation that medication instructions appear frequently in our data set, we incorporated medication features by (1) using a medication information extraction system [69] to extract medications and its related attributes (eg, drug name, dosage, duration, form, frequency, route, and strength) from the text and (2) converting the extracted attributes into composite features. We also incorporated additional features shown to be useful in the previous 2018 ClinicalSTS challenge, including domain-specific features and phrasal similarity features. Details on these features are provided in Multimedia Appendix 3 [50,51,69-71].

### Ensemble Methods

A total of 3 learning algorithms for regression were used for ensembling language model outputs and features: linear regression, Bayesian regression, and ridge regression. Note that we also explored random forest and XGBoost, which were used in the previous year’s winning systems, but found that they underperformed, and therefore, we did not use those methods. On the basis of the performance on the internal test data set, we experimented with incrementally averaging different combinations of the constituent model outputs while adding the other similarity features previously described. A detailed explanation of the training parameters is provided in Multimedia Appendix 2.

**Figure 4:**
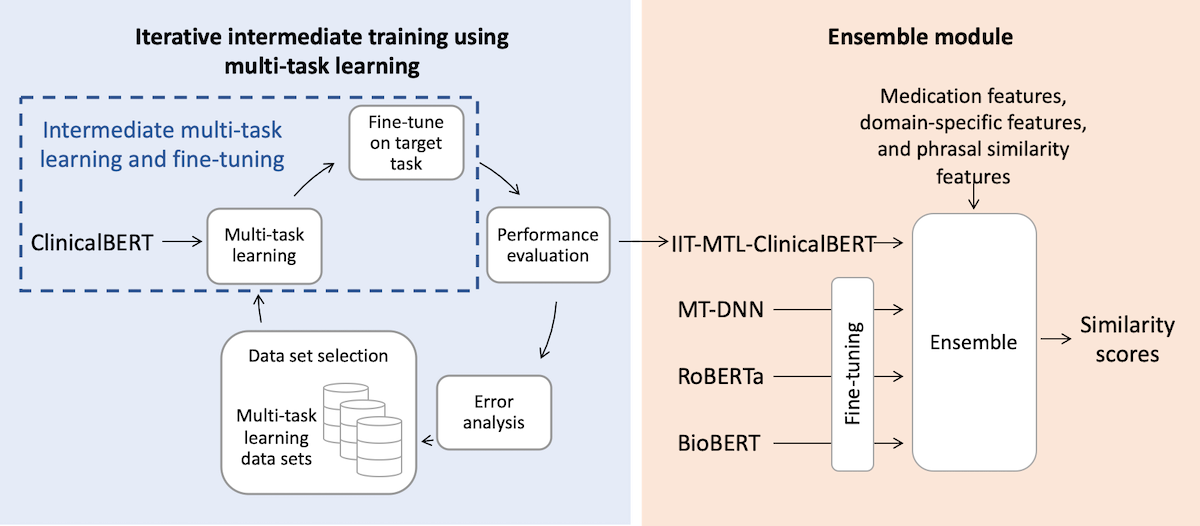
Overview of our end-to-end system. ClinicalBERT: bidirectional encoder representations from transformers on clinical text; IIT-MTL-ClinicalBERT: iterative intermediate training using multi-task learning on ClinicalBERT; MT-DNN: multi-task deep neural networks; RoBERTa: robustly optimized BERT approach; BioBERT: bidirectional encoder representations from transformers for biomedical text mining.

Figure 4 presents an overview of our end-to-end system on the ClinicalSTS task, consisting of an iterative intermediate multi-task training step followed by an ensemble module. Note that the intermediate MTL and fine-tuning portion of Figure 4 was presented earlier in more detail in Figure 3.

### Evaluation Metrics

We evaluated the proposed system using the evaluation script released by the organizers of the 2019 n2c2/OHNLP challenge to measure the Pearson correlation coefficient (PCC) between the human-annotated (gold standard) and predicted clinical semantic similarity scores. In the Results section, we report the PCC on the internal test data set for each iteration in IIT-MTL as well as on each combination of language models tried during ensembling. We also report the PCC for our 3 official submissions to the 2019 n2c2/OHNLP challenge on both the internal test data set and withheld external test data set.

## Results

### Iterative Intermediate Training Using MTL

Table 4 presents the results of each iteration in IIT-MTL. In comparison with the ClinicalBERT baseline, the addition of complementary data sets improved the overall model performance. Notably, not all data set additions resulted in improved performance. This is highlighted in iteration 5, where the addition of QQP led to a significant drop in performance. As the model from iteration 4 showed the best performance on the internal test data set, we adopted this variant for the final IIT-MTL-ClinicalBERT model.

**Table 4:** Results of each iteration of iterative intermediate training using multi-task learning.

Experiment and language model		Data sets used for iterative intermediate training approach using multi-task learning						Pearson correlation coefficient on internal test
		STS-B^a^	RQE^b^	MedNLI^c^	Topic	MedNER^d^	QQP^e^	
**BL^f^**								
	1 BERT^g^	—^h^	—	—	—	—	—	0.834
	2 ClinicalBERT^i^	—	—	—	—	—	—	0.848
**Iter^j^**								
	1 ClinicalBERT	✓^k^	—	—	—	—	—	0.852
	2 ClinicalBERT	✓	✓	✓	—	—	—	0.862
	3 ClinicalBERT	✓	✓	✓	✓	—	—	0.866
	4 ClinicalBERT	✓	✓	✓	✓	✓	—	*0.870* ^l^
	5 ClinicalBERT	✓	✓	✓	✓	✓	✓	0.856

^a^STS-B: semantic textual similarity benchmark.

^b^RQE: Recognizing Question Entailment.

^c^MedNLI: Natural Language Inference data set for the clinical domain.

^d^MedNER: Medication-NER data set.

^e^QQP: Quora Question Pair data set.

^f^BL: baseline.

^g^BERT: bidirectional encoder representations from transformers.

^h^Indicates data set was not used for this experiment.

^i^ClinicalBERT: bidirectional encoder representations from transformers on clinical text mining.

^j^Iter: iteration.

^k^Indicates data sets that were trained together in multi-task learning.

^l^Italics signify highest Pearson correlation coefficient obtained on internal test data set.

### Ensemble Module

Table 5 presents the results of the language model ensemble experiments performed on the internal test data set. Here, the statistical mean of the normalized language model outputs was used as our ensemble method. Of the individual models, IIT-MTL-ClinicalBERT and BioBERT, which were pretrained on clinical and biomedical corpora, respectively, achieved higher PCC as compared with MT-DNN and RoBERTa, which were pretrained only on general domain corpora. In general, ensembled models performed better than the individual constituent models alone, with the combination of IIT-MTL-ClinicalBERT, BioBERT, and MT-DNN resulting in the highest performance (PCC 0.8809) on the internal test data set.

**Table 5:** Ablation study of language models utilized in the ensemble module. The statistical mean of the language model outputs was used as the ensembling method.

Experiment	Language model ensemble				Pearson correlation coefficient on internal test
	IIT-MTL-ClinicalBERT^a^	BioBERT^b^	MT-DNN^c^	RoBERTa^d^	
1	✓^e^	—^f^	—	—	0.8711
2	—	✓	—	—	0.8707
3	—	—	✓	—	0.8685
4	—	—	—	✓	0.8578
5	✓	✓	—	—	0.8754
6	—	✓	✓	—	0.8780
7	—	—	✓	✓	0.8722
8	✓	—	—	✓	0.8741
9	✓	—	✓	—	0.8796
10	—	✓	—	✓	0.8720
11	✓	✓	✓	—	*0.8809* ^g^
12	—	✓	✓	✓	0.8769
13	✓	—	✓	✓	0.8787
14	✓	✓	—	✓	0.8764
15	✓	✓	✓	✓	0.8795

^a^IIT-MTL-ClinicalBERT: iterative intermediate training using multi-task learning on ClinicalBERT.

^b^BioBERT: bidirectional encoder representations from transformers for biomedical text mining.

^c^MT-DNN: multi-task deep neural networks.

^d^RoBERTa: robustly optimized bidirectional encoder representations from transformers approach.

^e^Indicates which language models are included in the ensemble.

^f^Indicates language model was not used for this experiment.

^g^Italics signify the highest Pearson correlation coefficient obtained on internal test data set.

On the basis of the experiments presented in Table 5, IIT-MTL-ClinicalBERT & BioBERT & MT-DNN was adopted as the base combination of language models for our official submissions. Table 6 presents the results of this base combination of language models, with incremental addition of other similarity features using four different ensemble methods. Results are shown for both the internal and withheld external test data sets. Note that the addition of domain-specific and phrasal similarity features has been included in Table 6 for completeness (although it resulted in lower performance) because it was part of our official submissions.

**Table 6:** End-to-end ensemble module and official submission results.

Components	Pearson correlation coefficient on internal test^a^					Pearson correlation coefficient on external test^a^			
	Mean	LR^b^	BR^c^	RR^d^	Mean		LR	BR	RR
IIT-MTL-ClinicalBERT^e^ & MT-DNN^f^ & BioBERT^g^	*0.8809*	0.8796	0.8795	0.8796	*0.9006*		0.8978	0.8978	0.8978
+ medication features	N/A^h^	*0.8841*	0.8832	0.8831	N/A		*0.9010*	0.8997	0.8975
+ domain-specific and phrasal similarity features	N/A	0.8733	0.8741	*0.8799*	N/A		0.8861	0.8920	*0.8875*

^a^Italics signify the Pearson correlation coefficient obtained on the internal and external test data set corresponding to the three configurations (components and ensemble method) that were our official submissions to the 2019 n2c2/OHNLP challenge.

^b^LR: linear regression.

^c^BR: Bayesian regression.

^d^RR: ridge regression.

^e^IIT-MTL-ClinicalBERT: iterative intermediate training using multi-task learning on ClinicalBERT.

^f^MT-DNN: multi-task deep neural networks.

^g^BioBERT: bidirectional encoder representations from transformers for biomedical text mining.

^h^N/A: not applicable.

### Official Submission

The best performing configurations on the internal test data set, as shown in Table 6, were entered as our official submissions to the 2019 n2c2/OHNLP ClinicalSTS challenge. The details of each of our 3 official submissions are as follows:

- Submission 1: IIT-MTL-ClinicalBERT & MT-DNN & BioBERT
  A statistical mean of the scores produced by the language models, specifically IIT-MTL-ClinicalBERT, MT-DNN, and BioBERT.
- Submission 2: IIT-MTL-ClinicalBERT & MT-DNN & BioBERT+medication features
  A linear regression model trained on each component output from Submission 1 and medication features.
- Submission 3: IIT-MTL-ClinicalBERT & MT-DNN & BioBERT+medication features+domain-specific and phrasal similarity features
  A ridge regression model trained on all features from Submission 2 and phrasal similarity and domain-specific features.

Our submission 2 achieved first place out of 87 submitted systems with a PCC of 0.9010 based on the official results. Our submission 1 achieved second place with a PCC of 0.9006.

With the release of the external test data set, we reran the experiments for language model ensembling on the external test data set. We identified the highest performing configuration on the external test data set as the statistical mean of the scores produced by the combination of IIT-MTL-ClinicalBERT, MT-DNN, and RoBERTa, which resulted in a PCC of 0.9025.

## Discussion

### Principal Findings

Iterative intermediate training using MTL is an effective way to leverage annotated data from related tasks to improve performance on the target task. However, it is critical to select data sets that can induce contextualized embeddings necessary for the target task. If the network is tasked with making predictions on unrelated tasks, negative transfer may ensue, resulting in lower quality predictions on the target task. Applying IIT-MTL to train ClinicalBERT with related tasks—STS-B, RQE, MedNLI, Topic, and MedNER—resulted in improved performance on the target ClinicalSTS task. However, the addition of QQP to the MTL step resulted in a significant drop in performance. This may be attributed to the fact that, in contrast to the other data sets used, QQP was created for a different sentence pair task (classification rather than regression) on the general domain (as opposed to RQE and MedNLI, which are on the clinical domain). This illustrates the importance of data set selection for the effectiveness of the intermediate multi-task training step.

Ensembling language models pretrained on domain-specific and domain-independent corpora incorporates different aspects of clinical semantic similarity. Table 7 presents the ground truth for two sentence pairs, along with predictions from each constituent model. The first sentence pair contains minimal domain-specific terminology; hence, the models trained on domain-independent corpora, MT-DNN and RoBERTa, predicted scores closer to the ground truth. The low ground truth score in the second sentence pair is because of dissimilar clinical concepts within the text; hence, the models trained on domain-specific corpora, IIT-MTL-ClinicalBERT and BioBERT, predicted scores closer to the ground truth.

**Table 7:** Sample sentence pairs with ground truth annotations and predictions from three language models used in the final ensembled system.

Sentence 1	Sentence 2	Ground Truth	Predictions			
			IIT-MTL-ClinicalBERT^a^	BioBERT^b^	MT-DNN^c^	RoBERTa^d^
“The following consent was read to the patient and accepted to order testing.”	“We explained the risks, benefits, and alternatives, and the patient agreed to proceed.”	2.5	0.61	1.01	2.15	2.51
“Negative for coughing up blood, coughing up mucus (phlegm) and wheezing.”	“Negative for abdominal pain, blood in stool, constipation, diarrhea and vomiting.”	0.5	1.04	1.18	2.34	1.74

^a^IIT-MTL-ClinicalBERT: iterative intermediate training using multi-task learning on ClinicalBERT.

^b^BioBERT: bidirectional encoder representations from transformers for biomedical text mining.

^c^MT-DNN: multi-task deep neural networks.

^d^RoBERTa: robustly optimized bidirectional encoder representations from transformers approach.

### Analysis of Model Performance

Our best official submission achieved a PCC of 0.9010 on the external test data set. However, the model performance varies significantly depending on the gold similarity scores. On the low and high ends of the gold scores, [0-2) or [4-5], our model achieves a PCC of 0.9234. However, in the middle range of the gold scores, [2-4), it performs much worse with a PCC of 0.5631. The lower performance in the middle range can be partially attributed to ground truth issues. Weak-to-moderate interannotator agreement (0.6 weighted Cohen kappa) coupled with the lack of an adjudication process (scores from 2 annotators were averaged to provide the gold score), led to concentration of annotation errors in the middle range of the gold scores. For example, greater disagreement between 2 annotators (eg, gold scores 1 and 5) will end up in the middle range (final averaged score 3) as compared with low disagreements (eg, 4 and 5 with the final score of 4.5). The drop in performance in the middle range may also indicate that although our model performs well at distinguishing completely similar or dissimilar sentence pairs, it struggles in scoring sentences with moderate clinical semantic similarity.

To further investigate this behavior, we studied how predictions varied for each similarity interval using the withheld external test data set. For this, we converted the continuous range gold scores and our model predictions into 5 intervals: [0,1), [1-2), [2-3), [3-4), [4-5]. Using these intervals, we then calculated the F1-score by computing true positives, false positives, and false negatives. A prediction is a true positive if the gold score is in the same similarity interval as the prediction; otherwise, it is termed as false positive (in the predicted interval) and false negative (in the gold interval). Our best model achieves a relatively high F1-score at the extreme ranges (0.77, 0.80, and 0.71 for [0,1), [1-2), [4-5], respectively) but struggles in the middle intervals (0.23 and 0.44 for [2-3) and [3-4), respectively).

### Limitations and Future Work

We acknowledge certain limitations of this study. First, these results are specific to the 2019 n2c2/OHNLP ClinicalSTS data set, which contains clinical text snippets from a single EHR data warehouse (Mayo Clinic EHR data warehouse). Furthermore, the chosen sentence pairs have high surface lexical similarity (ie, candidate pairs must have ≥0.45 average score of Ratcliff/Obershelp pattern matching algorithm, cosine similarity, and Levenshtein distance), which limits the variation in the data set. Thus, there is a need to validate this process on a more diverse ground truth, which (1) contains clinical text from multiple data warehouses and (2) allows for a less restrictive sentence pairing. Second, we observed inconsistencies in the ground truth, which may be inherent to a complex task such as clinical semantic textual similarity. We have made preliminary progress in quantifying these errors and their impact on the results, but more work is needed in this direction. Finally, although our system has achieved high PCC on the ClinicalSTS task, additional research is still needed to understand how to apply this foundational task to the real-world problem of bloated, disorganized clinical documentation.

Although our system achieved state-of-the-art results in the challenge, the proposed system has following avenues for improvement and further exploration:

1. The data set selection process in IIT-MTL is largely manual, driven by empirical observations and domain knowledge. Recent developments in automatic machine learning (AutoML), ranging from optimizing hyper-parameters using random search [72] to discovering novel neural architectures using reinforcement learning [73], have shown promising results. We plan to explore AutoML to relieve this manual effort in the future.
2. The language model ensemble works well for inducing domain-specific and domain-independent knowledge. However, this process remains largely intuitive. We plan to explore how language modeling objectives influence the domain adaptability of the learned language models on the target task.
3. At the time of the challenge, we applied our IIT-MTL methodology only to ClinicalBERT because of time constraints. We plan to employ our IIT-MTL methodology on other implemented language models and evaluate their performance.
4. Our proposed system has a significant computational cost, as we leverage several transformer-based language models. We plan to explore the performance impact of replacing these models with their less computationally expensive counterparts [74].
5. In our experiments, inclusion of domain-specific and phrasal features led to a drop in performance. This is likely because of effective learning of these features by pretrained transformer-based language models, as observed in the general domain [75,76]. We wish to investigate this behavior further by utilizing probing tasks [77] in transformer language models.

### Conclusions

In this study, we presented an effective methodology leveraging (1) an iterative intermediate training step in a MTL setup and (2) multiple language models pretrained on diverse corpora, which achieved first place in the 2019 ClinicalSTS challenge. This study demonstrates the potential for IIT-MTL to improve the performance of other tasks restricted by limited data sets. This contribution opens new avenues of exploration for optimized data set selection to generate more robust and universal contextual representations of text in the clinical domain.

## Appendix

Multimedia Appendix 1 Data sets used in iterative intermediate training approach using multi-task learning methodology.

Multimedia Appendix 2 Experimental settings.

Multimedia Appendix 3 Implementation details of other similarity features.

## Abbreviations

BERT: bidirectional encoder representations from transformers
BioBERT: bidirectional encoder representations from transformers for biomedical text mining
ClinicalBERT: bidirectional encoder representations from transformers on clinical text mining
ClinicalSTS: clinical semantic textual similarity
EHR: electronic health record
IIT-MTL-ClinicalBERT: iterative intermediate training using multi-task learning on ClinicalBERT
IIT-MTL: iterative intermediate training approach using multi-task learning
MedNER: medication named entity recognition data set
MedNLI: natural language inference data set for the clinical domain
MIMIC-III: Medical Information Mart for Intensive Care III
MT-DNN: multi-task deep neural networks
MTL: multi-task learning
n2c2: National Natural Language Processing Clinical Challenges
NLP: natural language processing
OHNLP: Open Health Natural Language Processing Consortium
PCC: Pearson correlation coefficient
PMC: PubMed Central
QQP: Quora question pairs
RoBERTa: robustly optimized bidirectional encoder representations from transformers approach
RQE: recognizing question entailment
STS-B: semantic textual similarity benchmark
STS: semantic textual similarity
