# Supplementary material for: Identification of Semantically Similar Sentences in Clinical Notes: Iterative Intermediate Training Using Multi-Task Learning
Source: JMIR Med Inform. 2020 Nov 27;8(11):e22508. doi: 10.2196/22508 (PMC7732709; doi:10.2196/22508)
Supplement: Multimedia Appendix 1 [file medinform_v8i11e22508_app1.pdf]

Multimedia Appendix 1: Data sets used in iterative intermediate training approach using multi-task learning methodology

Data sets

We utilized several sizeable annotated data sets during our intermediate multi-task training step. We describe the data sets in detail below:

STS-B: The Semantic Textual Similarity Benchmark [1] data set is a collection of sentence pairs drawn from news headlines, user forums and image captions. The task is to predict a continuous similarity score from 0 to 5, where 0 means no semantic similarity and 5 means complete semantic equivalence. This is a general domain data set for the STS task and contains 8,628 sentence pairs.

RQE: The Recognizing Question Entailment (RQE) data set is a collection of 8,588 medical question pairs created by [2] using a collection of existing Question-Answer Pairs from [3]. Given a pair of questions (Q1, Q2), this is a binary classification task, where the goal is to identify if the meaning of one question (Q2) can be inferred (entailed) from the other question (Q1).

MedNLI: The MedNLI [4] data set is a collection of 14,049 sentence pairs addressing the natural language inference task (NLI) in the clinical domain. Given a hypothesis-premise pair (H,P), where H is a physician description of the patient's condition (true or false), and P is a sentence extracted from a clinical note in the MIMIC-III corpus, the task is to predict the relation between two sentences, namely entailment, neutral or contradiction.

Topic: This data set is created on a randomly sampled set of 1300 sentences derived from sentence pairs in the ClinicalSTS [5] task. During our analysis of the ClinicalSTS data, we found that sentences belong to a fixed set of topics namely, SDOH (social determinants of health, e.g. "The patient states they cannot complete the following tasks on their own: Housekeeping, using transportation and managing medications."), MED (medication instructions, e.g. "Prednisone 20 mg tablet 3 tablets by mouth one time daily."), SIGNORSYMPTOM (sign or symptoms, e.g. "Musculoskeletal: Positive for gait problem, joint swelling and extremity pain."), EXPLAIN (a concise explanation of the clinical visit e.g. "Discussed the risks, benefits, alternatives and the necessity of other members of the healthcare team participating in the procedure.") or OTHER. This is used as a sentence classification data set, where the task is to predict a topic label for an input sentence.

MedNER: We automatically generate the MedNER data set, by annotating a random sample of 1000 MIMIC-III [6] clinical notes using a medication extraction tool [7], with labels such as *medications*, and its associated attributes, such as *strength*, *form*, *frequency*, *route*, *dosage* and *duration*.

QQP: The Quora-Question Pair data set [8] extracted from the actual Quora data consists of 404,290 question pairs, and the task is to determine if the two questions are duplicates of each

other i.e. they are completely semantically equivalent. This is a binary classification task, where duplicate question pairs are labeled as 1, and others are labeled as 0.

References

1. Cer D, Diab M, Agirre E, Lopez-Gazpio I, Specia L. SemEval-2017 Task 1: Semantic Textual Similarity Multilingual and Crosslingual Focused Evaluation. 2017. [doi: 10.18653/v1/s17-2001]
2. Abacha A Ben, Dina DF. Recognizing Question Entailment for Medical Question Answering. AMIA . Annu Symp proceedings AMIA Symp 2016;
3. Ely JW, Osheroff JA, Gorman PN, Ebell MH, Chambliss ML, Pifer EA, Stavri PZ. A taxonomy of generic clinical questions: Classification study. Br Med J 2000; PMID:10938054
4. Romanov A, Shivade C. Lessons from natural language inference in the clinical domain. Proc 2018 Conf Empir Methods Nat Lang Process EMNLP 2018 2018. p. 1586–1596. [doi: 10.18653/v1/d18-1187]
5. Yanshan Wang, Sunyang Fu, Feichen Shen, Sam Henry, Ozlem Uzuner HL. JMIR Medical Informatics #23375: Overview of the 2019 n2c2/OHNLP Track on Clinical Semantic Textual Similarity. JMIR Med Informatics [Internet] 2020 [cited 2020 Oct 12]; Available from: https://medinform.jmir.org/preprint/23375
6. Johnson AEW, Pollard TJ, Shen L, Lehman LWH, Feng M, Ghassemi M, Moody B, Szolovits P, Anthony Celi L, Mark RG. MIMIC-III, a freely accessible critical care database. Sci Data 2016;3:1–9. PMID:27219127
7. Xu H, Stenner SP, Doan S, Johnson KB, Waitman LR, Denny JC. MedEx: A medication information extraction system for clinical narratives. J Am Med Informatics Assoc 2010; PMID:20064797
8. Quora Question Pairs | Kaggle [Internet]. [cited 2020 Nov 2]. Available from: https://www.kaggle.com/c/quora-question-pairs
