# Supplementary material for: Identification of Semantically Similar Sentences in Clinical Notes: Iterative Intermediate Training Using Multi-Task Learning
Source: JMIR Med Inform. 2020 Nov 27;8(11):e22508. doi: 10.2196/22508 (PMC7732709; doi:10.2196/22508)
Supplement: Multimedia Appendix 2 [file medinform_v8i11e22508_app2.pdf]

Multimedia Appendix 2: Experimental settings

Experimental settings

For our experiments, we split our training dataset (1642 examples) into 75%, 15% and 10% to form our train (1232 examples), validation (246 examples) and internal test dataset (164 examples). Hyperparameters are tuned on the validation dataset.

Experimental settings for multi-task architecture

Our implementation of Multi-task architecture is based on [1][2]. We trained our model on two NVIDIA(R) V100 GPU using the PyTorch framework. As we are using BERT-base architecture, all the texts were tokenized using WordPieces[3] and tokenized text were chopped to spans no longer than 512 tokens. We used Adamax [4] as our optimizer with a learning rate of 5e-5 and a batch size of 32 by following [5]. The maximum number of epochs (epochmax) was set to 100. A linear learning rate decay schedule with warm-up over 0.1 was used. To avoid the exploding gradient problem, we clipped the gradient norm within 1. We use the same hyperparameters for all the task heads. The hyperparameters are summarized in Table 1.

Table 1. Hyperparameters for multi-task learning.

**Hyperparameter**	**Value**
Learning Rate	5e-5
Batch Size	32
$epoch^{max}$	100
Dropout	0.1
Optimizer	Adamax

Experimental settings for fine-tuning

To fine-tune the IIT-MTL-ClinicalBERT on specific tasks, we change the maximum number of epochs to 10 and learning rate to 1e−5. All the hyperparameters are summarized in Table 2.

Table 2. Hyperparameters for Fine-tuning.

**Hyperparameter**	**Value**
Learning Rate	1e-5
Batch Size	32
$epoch^{max}$	10
Dropout	0.1
Optimizer	Adamax

Experimental settings for the ensemble module

Table 3 provides the parameters used for Bayesian regression and ridge regression.

Table 3. Experimental settings for Bayesian regression and ridge regression.

Technique	Parameter	Value
Bayesian Regression	Number of iterations	300
	alpha_1[a]	1.15e-06
	alpha_2[b]	1.02e-06
	lambda_1[c]	1e-06
	rate[d]	1e-06
Ridge Regression	alpha	1
	solver	auto[e]

[a] shape parameter for the Gamma distribution prior over the alpha parameter
[b] rate parameter for the Gamma distribution prior over the alpha parameter
[c] shape parameter for the Gamma distribution prior over the lambda parameter
[d] parameter for the Gamma distribution prior over the lambda parameter
[e] chooses the solver automatically based on the type of data

References

1. Liu X, He P, Chen W, Gao J. Multi-task deep neural networks for natural language understanding. ACL 2019 - 57th Annu Meet Assoc Comput Linguist Proc Conf 2019;4487–4496.
2. namisan/mt-dnn: Multi-Task Deep Neural Networks for Natural Language Understanding [Internet]. [cited 2020 Nov 2]. Available from: https://github.com/namisan/mt-dnn
3. [61] Wu Y, Schuster M, Chen Z, Le Q V., Norouzi M, Macherey W, Krikun M, Cao Y, Gao Q, Macherey K, Klingner J, Shah A, Johnson M, Liu X, Kaiser Ł, Gouws S, Kato Y, Kudo T, Kazawa H, Stevens K, Kurian G, Patil N, Wang W, Young C, Smith J, Riesa J, Rudnick A, Vinyals O, Corrado G, Hughes M, Dean J. Google's Neural Machine Translation System: Bridging the Gap between Human and Machine Translation. 2016;1–23. Available from: http://arxiv.org/abs/1609.08144
4. Kingma DP, Ba JL. Adam: A method for stochastic optimization. 3rd Int Conf Learn Represent ICLR 2015 - Conf Track Proc 2015.
5. Devlin J, Chang MW, Lee K, Toutanova K. BERT: Pre-training of deep bidirectional transformers for language understanding. NAACL HLT 2019 - 2019 Conf North Am Chapter Assoc Comput Linguist Hum Lang Technol - Proc Conf 2019;1(Mlm):4171–4186.
