# Supplementary material for: Identification of Semantically Similar Sentences in Clinical Notes: Iterative Intermediate Training Using Multi-Task Learning
Source: JMIR Med Inform. 2020 Nov 27;8(11):e22508. doi: 10.2196/22508 (PMC7732709; doi:10.2196/22508)
Supplement: Multimedia Appendix 3 [file medinform_v8i11e22508_app3.pdf]

Multimedia Appendix 3: Implementation details of other similarity features

Medication features

We incorporated medication features by using a medication information extraction system [1] to extract medications and its related attributes (e.g. drug name, dosage, duration, form, frequency, route, strength) from the text and then, (2) converting the extracted attributes into composite features. We compare each extracted medication attribute type between the two sentences, and create the composite feature based on presence/absence of these attributes as follows:

- Feature1: If the attribute type is present in both sentences, and the attribute text matches
- Feature2: If the attribute type is present in both sentences, and the attribute text is different
- Feature3: If the attribute type is present in only one sentence
- Feature4: If the attribute type is absent from both sentences

We further employ grid search to tune our hyper-parameters for each of these features, which gave us values 0.9, -1.0, -2.8, and 0.5 for features 1-4 respectively.

Domain-specific features and phrasal similarity features

We also incorporated additional features shown to be useful in the previous 2018 ClinicalSTS challenge, including domain-specific features and phrasal similarity features. Domain-specific features were derived using the Unified Medical Language System (UMLS) [2] semantic type match and concept unique identifier relation inference chains (e.g. Medication A contains Medication B treats Disease C is a Disease D). Phrasal similarity features were generated leveraging WordNet synonym/antonym [3] and alignment-based scores [4,5].

References

1. Mahajan D, Liang JJ, Tsou C-H. Extracting Daily Dosage from Medication Instructions in EHRs: An Automated Approach and Lessons Learned. arXiv:200510899 2020;
2. Lindberg DAB, Humphreys BL, McCray AT. The unified medical language system. Methods Inf Med. 1993. [doi: 10.1055/s-0038-1634945]
3. Miller GA. WordNet: A Lexical Database for English. Commun ACM 1995; [doi: 10.1145/219717.219748]
4. Sultan MA, Bethard S, Sumner T. DLS$@$CU: Sentence Similarity from Word Alignment. 2015. [doi: 10.3115/v1/s14-2039]
5. Sultan MA, Bethard S, Sumner T. DLS$@$CU: Sentence Similarity from Word Alignment and Semantic Vector Composition. 2015. [doi: 10.18653/v1/s15-2027]
